# Altered Circulating MicroRNA Profiles After Endurance Training: A Cohort Study of Ultramarathon Runners

**DOI:** 10.3389/fphys.2021.792931

**Published:** 2022-01-25

**Authors:** Ceren Eyileten, Zofia Wicik, Alex Fitas, Mikolaj Marszalek, Jenny E. Simon, Salvatore De Rosa, Szczepan Wiecha, Jeffrey Palatini, Marek Postula, Lukasz A. Malek

**Affiliations:** ^1^Department of Experimental and Clinical Pharmacology, Centre for Preclinical Research and Technology, Medical University of Warsaw, Warsaw, Poland; ^2^Genomics Core Facility, Centre of New Technologies, University of Warsaw, Warsaw, Poland; ^3^Division of Cardiology, Department of Medical and Surgical Sciences, Magna Graecia University, Catanzaro, Italy; ^4^Department of Physical Education and Health in Biala Podlaska, Józef Pilsudski University of Physical Education in Warsaw, Biala Podlaska, Poland; ^5^Department of Epidemiology, Cardiovascular Disease Prevention and Health Promotion, National Institute of Cardiology, Warsaw, Poland

**Keywords:** microRNAs, miRNA, bioinformatics analysis, endurance sport, *in silico* prediction

## Abstract

**Background:**

Despite the positive effects of endurance training on the cardiovascular (CV) system, excessive exercise induces not only physiological adaptations but also adverse changes in CV system, including the heart. We aimed to evaluate the selected miRNAs expression based on bioinformatic analysis and their changes before and after an ultramarathon run.

**Materials and Methods:**

Cardiac tissue-specific targets were identified with the Tissue 2.0 database. Gene-gene interaction data were retrieved from the STRING app for Cytoscape. Twenty-three endurance athletes were recruited to the study. Athletes ran to completion (100 km) or exhaustion (52–91 km, median 74 km). All participants completed pre- and post-run testing. miRNAs expressions were measured both before and after the race.

**Results:**

Enrichment analysis of the signaling pathways associated with the genes targeted by miRNAs selected for qRT-PCR validation (miR-1-3p, miR-126, miR-223, miR-125a-5p, miR-106a-5p, and miR-15a/b). All selected miRNAs showed overlap in regulation in pathways associated with cancer, IL-2 signaling, TGF-β signaling as well as BDNF signaling pathway. Analysis of metabolites revealed significant regulation of magnesium and guanosine triphosphate across analyzed miRNA targets. MiR-1-3p, miR-125a-5p, miR-126, and miR-223 expressions were measured in 23 experienced endurance athletes, before and after an ultramarathon wherein athletes ran to completion (100 km) or exhaustion (52–91 km, median 74 km). The expressions of miR-125a-5p, miR-126, and miR-223 were significantly increased after the race (*p* = 0.007, *p* = 0.001, *p* = 0.014, respectively). MiR-1-3p expression post-run showed a negative correlation with the post-run levels of high-sensitivity C-reactive protein (hs-CRP) (*r* = −0.632, *p* = 0.003). Higher miR-1-3p expression was found in runners, who finished the race under 10 h compared to runners who finished over 10 h (*p* = 0.001). Post-run miR-125a-5p expression showed a negative correlation with the peak lactate during the run (*r* = −0.576, *p* = 0.019).

**Conclusion:**

Extreme physical activity, as exemplified by an ultramarathon, is associated with changes in circulating miRNAs’ expression related to inflammation, fibrosis, and cardiac muscle function. In particular, the negative correlations between miR-125a-5p and lactate concentrations, and miR-1-3p and hs-CRP, support their role in specific exercise-induced adaptation. Further studies are essential to validate the long-term effect of these observations.

## Introduction

Cardiovascular diseases (CVDs) are the leading cause of death globally, accounting for nearly 18 million deaths annually (World Health Organization, 2019). It is estimated that the majority of CVDs could be prevented by modifications of lifestyle including change of dietary habits and regular physical activity. Regular exercise of moderate (at least 150 min per week) to high intensity (75 min/week) is recommended by the European Society of Cardiology to reduce atherosclerotic CV risk (Pelliccia et al., 2020). Despite the positive effects of endurance training on the CV system, excessive exercise may induce not only physiological adaptations but also adverse changes in CV system, including the heart. Cardiac alterations comprise modifications of its structure, electrical activity, or function, toward a phenotype resembling pathological states (George et al., 2012).

MicroRNAs (miRNAs, miRs) are small, endogenous RNAs that form complex signaling networks responsible for regulating cell differentiation, development and homeostasis. MiRNAs are able to regulate gene expression on the post-transcriptional level suppressing or enhancing the degradation of messenger RNA (mRNA) (Ha and Kim, 2014; Eyileten et al., 2018, 2020; Pordzik et al., 2018, 2019; Sabatino et al., 2019; Gasecka et al., 2020; Jarosz-Popek et al., 2020; Soplinska et al., 2020; Wicik et al., 2020; Wolska et al., 2020; Zareba et al., 2020; Jakubik et al., 2021). MiRNAs take part in regulation of cell growth, cell differentiation, apoptosis, proliferation and they are thus involved in pathophysiology of cardiovascular pathology such as hypertrophy, inflammation, fibrosis and cardiomyocyte damage (Feinberg and Moore, 2016; Tahamtan et al., 2018; Ji et al., 2019; Soplinska et al., 2020; Jakubik et al., 2021). Circulating miRNAs are changed due to acute and endurance exercise and can be related in the adaptations to exercise. As it was reviewed before, previous studies assessed miRNA plasma levels in marathon runners and showed altered expression levels of some miRNAs after marathon race and their relation with standard fitness parameters (Mooren et al., 2014; Soplinska et al., 2020). Moreover, other studies documented correlations between miRNAs expression and cardiac injury markers such as troponin plasma levels, n-terminal b-type natriuretic peptide (NT-pro-BNP), or creatine kinase-MB (CK-MB) (Baggish et al., 2014). Therefore, these findings can suggest their potential use as biomarkers of adaptive changes in response to endurance exercise.

Several miRNAs were found to correlate with individual anaerobic lactate threshold (LT) (Mooren et al., 2014), which is defined as the exercise intensity above which blood lactate concentrations increase rapidly. LT is an indicator of endurance performance corresponding to low/moderate exercise in high-level endurance athletes (Garnacho-Castaño et al., 2015). Various evidence on the role of miRNAs in lactate dehydrogenase’s (LDH) activity exist, and it is postulated that some miRNAs may aggravate cell injury *via* LDH action enhancement (Ge et al., 2019). It is established that participation in ultramarathon runs leads to significant elevation in high-sensitivity troponin T (hs-TnT) concentration, although the mechanism of this phenomenon is poorly understood (Małek et al., 2020). Moreover, some miRNAs were found to be independently associated with the increase in hs-TnT levels (Widera et al., 2011).

Computational approaches are very useful in explaining the complex regulatory networks of miRNAs, and the identification of their functions and target genes in a cost-effective manner. Since each miRNA has multiple targets, it would be unrealistic to rely solely on laboratory experiments. Integrating *in silico* target prediction into the workflow is a powerful approach to orient the selection of promising molecules and enrich laboratory results with biological knowledge (Zhang et al., 2016).

Endurance intensive and long-term exercise is characterized by cardiovascular adaptation. Recently, the scarce clinical imaging studies on ultramarathon runners indicated that cardiovascular changes attributed to intensive training can resemble pathological states (Małek et al., 2021). Moreover, several molecular based studies showed that endurance training may increase inflammation and cardiac fibrosis (Parry-Williams and Sharma, 2020). We hypothesize that excessive endurance intensive exercise induces on a molecular basis pathological cardiac fibrosis, muscle hypertrophy and inflammation, which can come evident through the alterations of miRNA expression.

Therefore, we have aimed to perform bioinformatic analysis to determine the miRNAs related to angiogenesis, cardiac muscle function, muscle hypertrophy, coagulation, inflammation, and fibrosis processes based on detailed literature search which studied acute and chronic exercises. Results of bioinformatic analysis informed the selection of a panel of miRNAs that were then validated in a cohort of elite ultramarathon runners. Consequently, we aimed to evaluate the selected miRNAs expression changes before and after an ultramarathon run, as well as the association of miRNAs with hs-CRP and lactate concentrations in elite ultramarathon runners.

## Materials and Methods

### Bioinformatics Analysis MicroRNA Targets Prediction, Data Filtering, and Visualization as Interaction Networks

#### Article Search Process

Electronic databases PubMed and Scopus were searched up to January 2021. Original studies were reviewed based on: the clinical usefulness of miRNAs as novel biomarkers of adaptive alteration in response to endurance exercise, namely running and cycling based on human subjects. We also investigated review articles and meta-analyses, and their secondary references were examined for possible inclusion. Papers describing strength exercises were excluded from our analysis (Supplementary Table 1).

The following search syntax was used: “Search (“microRNAs” [MeSH Terms] OR “miR” [MeSH Terms] OR “miRNA” [MeSH Terms] OR “circulating miRNA” [MeSH Terms] OR “circulating microRNA” [MeSH Terms]) AND (“endurance training” [MeSH Terms] AND (“adaptation” [MeSH Terms] OR “change” [All Fields]) Filters: Humans”. Our search was limited to human studies and did not exclude studies on the basis of ethnicity.

#### Target Prediction

To identify targets of analyzed miRNAs we used multiMiR 1.4 R package (Ru et al., 2014). We searched the top 10% hits among all conserved and non-conserved target sites in 14 target prediction databases. For input miRNAs without mature versions of id’s we performed target predictions using all three combinations: stem-loop miRNA and -3p and -5p versions.

#### Selection of Cardiovascular Disease-Related Lists of Genes

To identify the genes associated with analyzed processes (angiogenesis, cardiac muscle functions, coagulation, fibrosis, hemopoiesis, inflammation, muscle hypertrophy, and platelet activity) among identified miRNAs targets we performed a screening of the Gene Ontology (GO) terms for the presence of key words using the biomaRt package in R (Durinck et al., 2009). GO terms associated with gene lists are available as Supplementary Table 2. In order to identify genes associated with CVD, we screened the DisgeNET database for this term and gene-disease associations (Piñero et al., 2020). Next, we selected genes which had at least five CVD related publications or three variants associated with CVD.

Tissue specific genes were selected by mining TISSUES2.0 database (Palasca et al., 2018). Genes which showed expression confidence score ≥ 1 (scale 0–5) in following tissues were used for further analysis: Atrium, Capillary pericyte, Cardiac muscle, Cardiac Purkinje_cell, Cardiac Purkinje fiber, Cardiofibroblast, Cardiomyoblast, Cardiovascular system, Heart endothelial cell, Heart, Heart ventricle, Left atrium, Left ventricle, and Pericyte.

#### Data Aggregation, Summarization, and Visualization

In order to aggregate and summarize miRNA-target interactions we used our wizbionet R package^1^ (Wicik et al., 2020, 2021; Walkiewicz et al., 2021). After aggregation we ranked the obtained results using clusterizer_oneR function. Clusterizer_oneR utilizes Jenks natural breaks optimization algorithm from the original OneR package as a non-arbitrary classification dividing numbers of regulated genes into four categories (clusters). Obtained results were sorted and visualized as heatmap using Morpheus software https://software.broadinstitute.org/morpheus/.

#### Enrichment Analysis

Enrichment analysis of the ontological terms associated with the targets of miRNAs selected for quantitative polymerase chain reaction (qPCR) validation was performed using EnrichR database API plugin and databases BioPlanet_2019, HMDB_Metabolites and Jensen_DISEASES datasets. In all analyses FDR corrected *p*-value cutoff was set as lower than 0.05. Visualization of the overlap between the enriched terms associated with the analyzed miRNAs was performed using https://software.broadinstitute.org/morpheus/.

### Study Group

The study was conducted on November 10, 2018 at the University of Physical Education in Warsaw.^2^ Ultramarathon runners did 65.10 laps of 1535.89 m distance on flat terrain (asphalt, bitumen track and short parts of cobblestone) which in total gave 100 km ultra-marathon run. The race was accredited by the Polish Athletics Association as the National Championships of 100 km. Total number of 23 healthy amateur runners (20 males) volunteered to participate in the study and to follow the whole protocol of the study. Initial screening was performed in case of each participant in the form of a medical questionnaire to exclude any known medical conditions as described previously (Małek et al., 2020). Blood was drawn from an antecubital vein to perform baseline analysis of hs-TnT and high sensitivity C-reactive protein (hs-CRP) levels. Simultaneously the analysis of baseline capillary lactate and glucose concentration was performed. Moreover, every six laps (approximately every 9.2 km) runners had fingertip capillary lactate and glucose assessment. The final assessment of capillary lactate and glucose concentration followed by venous blood draw for hs-TnT and hs-CRP was performed immediately after participants completed the run. In collaboration with a certified company (datasport.pl) we have collected data on the time of the race, together with the mean pace and total distance covered by each runner participating in the study.

### Blood Collection and Biomarker Measurement

Approximately 9 ml of blood was collected from an antecubital vein into a plasma separator tube just before and within 30 min after the race. The sample was kept at room temperature for 30 min prior to centrifugation at 1,500 × *g* for 15 min at 18–25°C. Plasma was aliquoted into 500 μl volumes and stored in −80°C freezer. In order to determine hs-CRP and hs-TnT, an electrochemiluminescence immunoassay method (ECLIA) Roche Cobas e411 analyser (Roche Diagnostics, Mannheim, Germany) was used. Reference values were set to <5 mg/dL and <14 ng/L, for hs-CRP and hs-TnT, respectively. Glucose and lactate assessment was carried out in intervals during the run through a fingertip capillary test with Biosen C-Line analyser (EKF Diagnostics, Cardiff, United Kingdom), as described previously (Małek et al., 2020).

### RNA Preparation, Detection, and Quantification of MicroRNAs by Quantitative Polymerase Chain Reaction

In order to purify samples from cell debris, plasma samples after thawing at room temperature were subjected to centrifugation at 16,000 × *g* for 10 min at 4°C. Total RNA was extracted using miRVana PARIS Kit (invitrogen, Applied technologies) from 500 μl of plasma. Subsequently, the obtained RNA template was subjected to a reverse transcription reaction using the TaqMan miRNA Reverse Transcription kit (ABI, CA, United States) according to guidelines provided by the manufacturer. Afterward, miRNA expressions were detected by qPCR using TaqMan miRNA Assay kits (ABI, CA, United States) for the corresponding miRNAs on a the CFX384 Touch Real-Time PCR Detection System (BioRad Inc., Hercules, CA, United States). Cel-miR-39 was added as an exogenous spike-in normalizer. Mean values of all reactions—performed in triplicate—were used in statistical analysis (De Rosa et al., 2017, 2018). MiRNA expressions are expressed as 2^–ΔCT^ (miRNA–cel-miR-39) (De Rosa et al., 2017, 2018), then log-transformed for statistical analysis.

### Statistical Analysis

All results for categorical variables were presented as a number and percentage. Continuous variables were expressed as mean ± standard deviation (SD) or median and interquartile range (IQR), depending on the normality of distribution assessed by means of the A Shapiro–Wilk test. The Student *t*-test or the Mann-Whitney test for unpaired samples, and Wilcoxon test for paired samples, were applied depending on the normality of the distribution. To assess the correlation between continuous variables, a Spearman test was applied. All tests were two-sided with the significance level of *p* < 0.05. Calculations were performed using SPSS version 22.0 (IBM Corporation, Chicago, IL, United States).

### Ethical Considerations

At the time of design, the study was congruent with the of the Declaration of Helsinki. Written informed consent form was obtained from all participants. Both the study protocol and the informed consent form were approved by the Ethics Committee of the Regional Medical Chamber in Warsaw (no. 52/17).

## Results

### Bioinformatic Analysis Results

**FIGURE 1:**
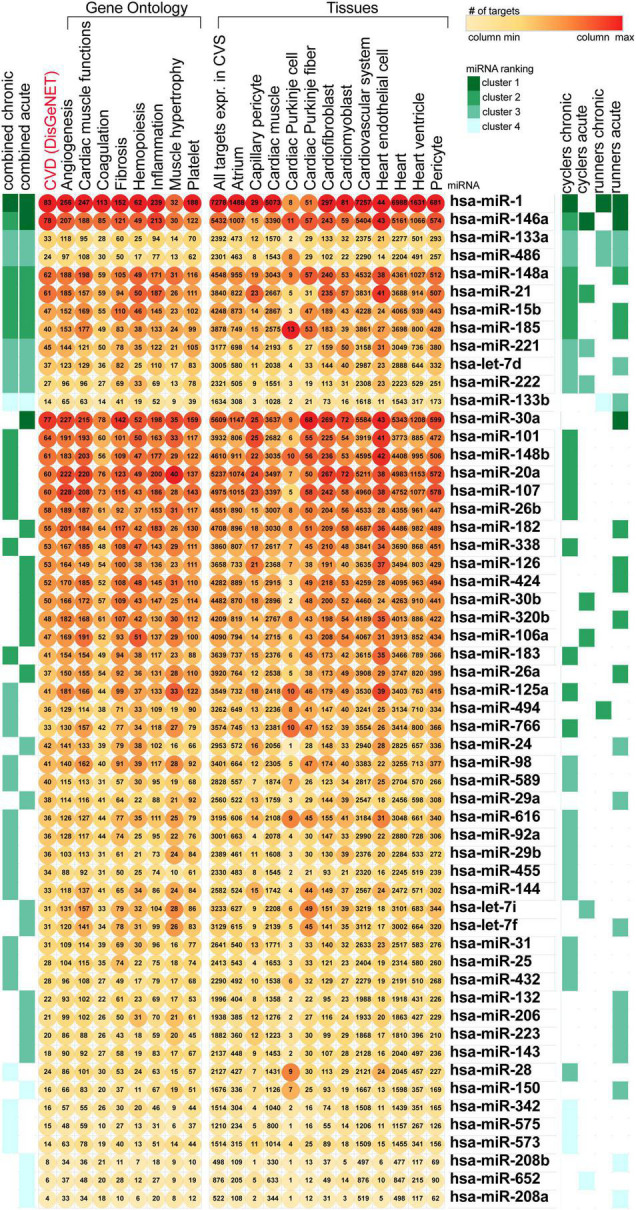
Ranking of the training-related miRNAs based on the number of targets associated with heart function. miRNA, miR, microRNA; CVS, cardiovascular system; CVD, cardiovascular disease.

To investigate miRNAs contribution to the type and intensity of endurance exertion, we conducted a bioinformatics analysis based on detailed literature search. We performed two simultaneous bioinformatics analyses: (i) tissue-specific and (ii) CV process-specific. We used as an input 55 miRNAs gathered from the literature, related to acute, and chronic exercises of runners and cyclers. We have ranked the miRNAs based on the number of process-related and tissue-related targets as presented in Figure 1. Our analysis didn’t identify any miRNA which would be uniquely associated with both chronic or both acute types of training. Thus rather we focused on identification of miRNAs the most affected by the physical activity.

Among the highly ranked miRNAs, miR-1 expression changes were related to most of the training programs and regulated the highest number of targets associated with the CV system. MiR-125a appeared in relation to chronic training programs, and targeted genes related to inflammation and CV system. Both miR-15b and miR-223 were found to be associated with acute phases of physical activity and were found in our previously published bioinformatic analysis as an important regulator of CV disease pathophysiology (Sabatino et al., 2019). Also, miR-126 expression responded to acute exposure of physical activity and regulated expression of targets related to capillary pericytes. Lastly, miR-106a was found to be influenced by acute training and had the highest number of targets related to cardiac muscle functions and haemopoesis. Therefore, miR-1-3p, miR-126, miR-223, miR-125a-5p, miR-106a-5p, and miR-15b were selected for the further qRT-PCR validation in our study. Moreover, although miR-15a did not appear in current bioinformatic analysis, we have included it to our qRT-PCR validation analysis as well, as miR-15a and miR-15b are in the same miRNA precursor family (miR-15) and was previously found by our team as a novel regulator of insulin signaling and glucose metabolism in bioinformatic analysis (Pordzik et al., 2019).

**FIGURE 2:**
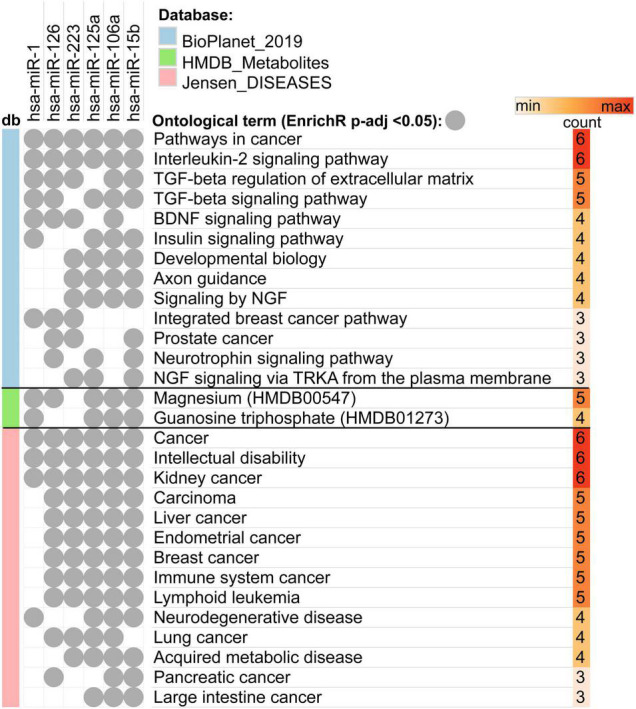
Overlap between enriched ontological terms (pathways, metabolites, and diseases) associated with genes targeted by miRNAs selected for qRT-PCR validation.

In order to identify the pathways regulated by the miRNAs selected for qRT-PCR validation (miR-1-3p, miR-126, miR-223, miR-125a-5p, miR-106a, and miR-15a/b) we performed enrichment analysis of the genes targeted by those miRNAs. All the selected miRNAs showed overlap in regulation in pathways associated with cancer, IL-2 signaling, TGF-β signaling as well as BDNF signaling pathway (Supplementary Figure 1). Analysis of metabolites revealed significant regulation of magnesium and guanosine triphosphate across analyzed miRNA targets. We also observed the highest overlap among the following diseases: Cancer, intellectual disability and kidney cancer (six miRNAs). Analyzed miRNAs also regulated genes associated with multiple other cancerous diseases including liver and endometrial cancer, as well as neurodegenerative and acquired metabolic disease (Figure 2).

### Participants

Twenty-three endurance athletes were recruited to the study. Athletes ran to completion (100 km) or exhaustion (52–91 km, median 74 km). All participants completed pre- and post-run testing. Detailed demographics are tabulated in Table 1 [also previously provided elsewhere (Małek et al., 2020)]. hs-CRP, lactate, hs-TnT and glucose levels for five runners were unavailable. The median running time in the studied group was 10.6 h (IQR 8.6–11.5) and the median pace was 8.7 min/km (IQR 8.0–9.4). Post-run values of blood pressure (both systolic and diastolic) and heart rate were significantly higher in comparison to pre-run values in all participants as expected (*p* = 0.0004, *p* < 0.0001, *p* < 0.0001, respectively). Importantly, hs-CRP values before the race were in normal range in all runners. On the other hand, hs-CRP levels significantly increased in all athletes after the race (*N* = 18; *p* < 0.0001) (Table 2), and they exceeded reference value in six cases (33%). Runners were divided into two different groups based on their post-run hs-CRP (cut off: ≤5 mg/dL) and maximum lactate concentration during the race (cut off: ≤4 mmol/L) concentration. Patients in the high hs-CRP group had significantly higher hs-TnT concentration changes (delta hs-TnT) compared to patients in the low hs-CRP group (*p* = 0.018).

**TABLE 1:** Baseline and running characteristics of the studied group.

Parameter	Ultra-marathon runners (*N* = 23)
Male sex (%)	20 (87)
Age, yrs (IQR)	45 (37–54)
BMI, kg/m2 (IQR)	24.7 (22.7–25.7)
Years of running (IQR)	4.5 (3.5–7.0)
Years of ultra running (IQR)	2 (0–3)
Weekly running distance, km (IQR)	65 (40–80)
Number of ultra races completed (IQR)	3 (0–10)
Longest completed race, km (IQR)	55 (42–80)

*Data are presented as number and percentage or median and IQR. BMI, Body mass index; IQR, Interquartile range; yrs, Years.*

**TABLE 2:** Pre- and post-run values of the analyzed parameters.

	Pre-run	Post-run	*P*
HR, bpm (*N* = 23)	54.5 (50–60)	81.5 (76–93)	**<0.0001**
SBP, mmHg (*N* = 18)	137 (130–146)	123 (109–133)	**0.0004**
DBP, mmHg (*N* = 18)	84 (92–91)	73 (70–78)	**<0.0001**
CRP, mg/dL (*N* = 18)	0.7 (0.43–1.1)	3.2 (1.9–8.1)	**<0.0001**
hs-TnT, ng/L (*N* = 18)	5 (3–7)	14 (12–26)	**<0.0001**
Lactate mmol/L (*N* = 18)	2 (1.7–2.4)	2.2 (1.4–3.5)	0.22
Glucose, mg/L (*N* = 18)	89 (86–95)	93 (80–100)	0.83

*bpm, Beats per minute; CRP, C-reactive protein; DBP, Diastolic blood pressure; HR, Heart rate; hs-TnT, High-sensitivity troponin T; SBP, Systolic blood pressure. P values marked with bold indicate statistically significant differences between the groups < 0.05.*

### Circulating MicroRNAs

**FIGURE 3:**
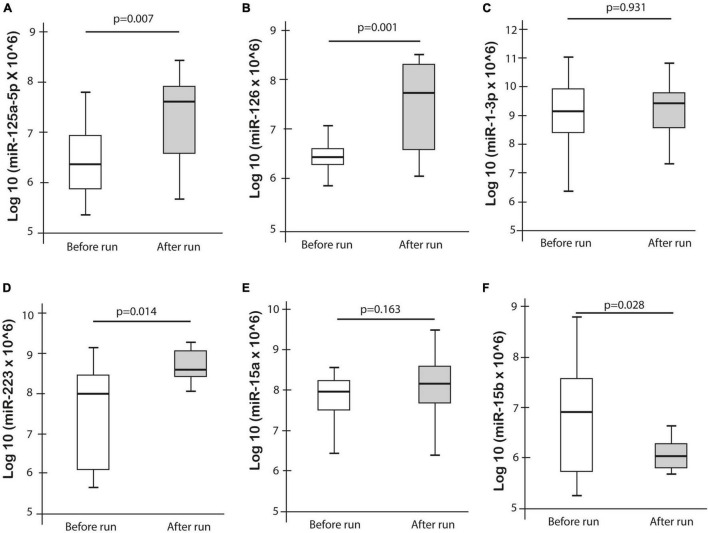
Differences in miRNA expression pre- and post-ultramarathon.

Expression levels of the selected miRNAs, miR-1-3p, miR-126, miR-223, miR-125a-5p, miR-106a-5p, and miR-15a/b were measured in 23 athletes in both pre- and post-ultramarathon run. The expression levels of miR-125a-5p (*p* = 0.007), miR-126 (*p* = 0.001), and miR-223 (*p* = 0.014) were significantly increased after the ultramarathon, whereas miR-15b was significantly decreased (*p* = 0.028). No significant difference was observed for miR-1-3p and miR-15a, while miR-106a was not detectable in any blood plasma sample (Figure 3).

### Correlations of MicroRNAs Expressions With Clinical Parameters and Race Duration

**FIGURE 4:**
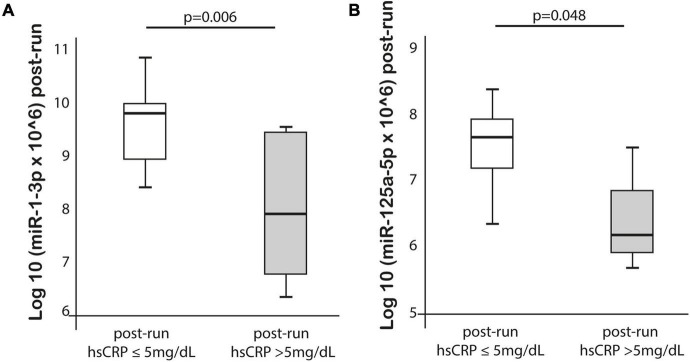
Differences in miR-1-3p and 125a-5p expression after completion the run in subgroups divided based on hs-CRP concentration.

**FIGURE 5:**
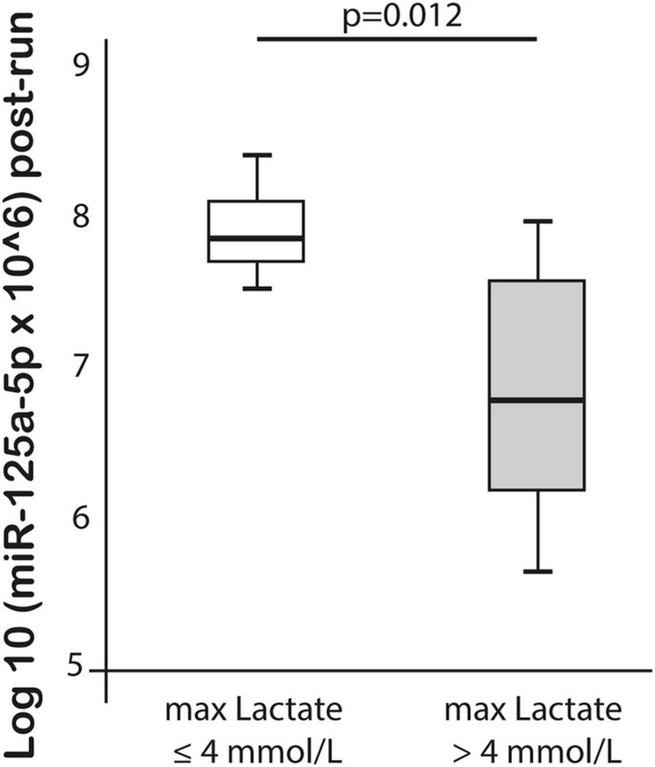
Difference in miR-125a-5p expression after completion of the run in subgroups divided based on lactate concentration.

**FIGURE 6:**
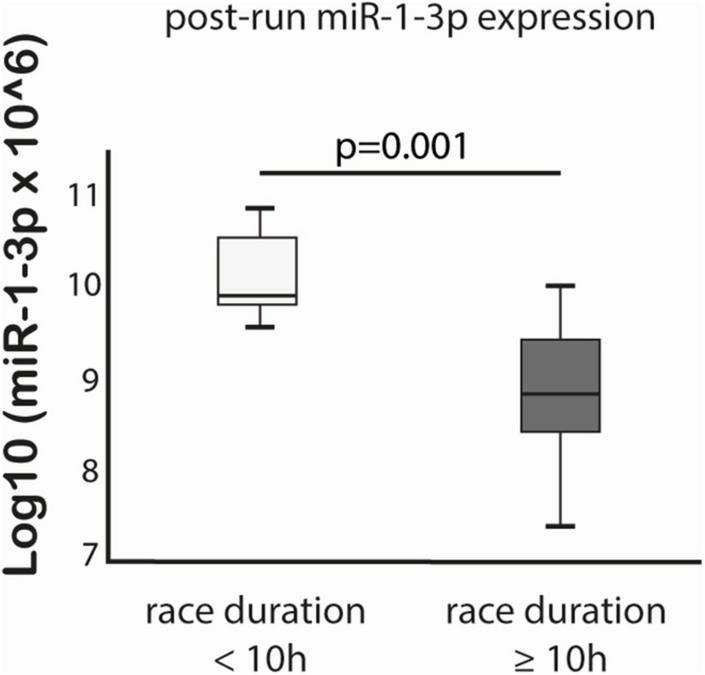
Difference in miR-1-3p expression after completion of the run in subgroups divided based on race duration cut-off 10 h.

MiR-125a-5p expression post-run was negatively correlated with max lactate levels during run (*r* = −0.576, *p* = 0.019) and miR-125a-5p pre-run showed positive correlation with delta hs-TnT levels (*r* = 0.807, *p* = 0.005). Moreover, miR-1-3p expression post-run was negatively correlated with hs-CRP levels post-run (*r* = −0.632, *p* = 0.003) (Table 3). MiR-1-3p (*p* = 0.006) and miR-125a-5p (*p* = 0.048) were significantly lower in post-run samples in the high hs-CRP group (Figure 4). Similarly, miR-125a-5p was found significantly lower (*p* = 0.012) in the high lactate group (Figure 5). MiR-1-3p was significantly lower in post-run samples in the runners who finished the race over 10 h (*p* = 0.001) (Figure 6). There were no correlations observed between miRNAs changes and changes of levels of biomarkers in ultramarathon runners (data not shown).

**TABLE 3:** Spearman’s correlation analysis.

miRNA	Parameter	*R* and *P*
miR-125a-5p pre-run	hs-TnT delta	*r* = 0.807, *p* = 0.005
miR-125a-5p post-run	max lactate (during the race)	*r* = −0.576, *p* = 0.019
miR-1-3p post-run	hs-CRP post-run	*r* = −0.632, *p* = 0.003
miR-15a post-run	Glucose post-run	*r* = −0.489, *p* = 0.019

## Discussion

Our study evaluated the effect of excessive physical fitness in a unique population of ultra-marathon runners on the expression changes of miRNAs associated with hemopoiesis, angiogenesis, cardiac muscle functions, and muscle hypertrophy selected on the basis of a bioinformatic analysis. Among the possible strategies to select miRNAs for validation studies, *in silico* bioinformatic analysis are beneficial, allowing to summon all available evidence and to generate predictions of specific targets and molecular interactions. Applying this framework, the current study is the first to identify the most relevant targets and to provide a validation on a very specific cohort of elite athletes. The results generated might be useful to establish new biomarkers of physiological adaptation in the endurance sportsman. Bioinformatics and computational analysis of data from systematic literature search highlighted the most promising circulating miRNAs modulated by physical exercise and endurance training. Taking into account that *in silico* analysis did not identify the miRNAs which would be specific only for chronic or acute types of training, we hypothesize that the difference in miRNAs profile is more related to the level of the miRNA, rather than its presence/absence. We confirmed that expressions of miR-125a-5p, miR-126, and miR-223 were significantly higher after the 100-km race in a cohort of 23 elite endurance sportsmen.

Moreover, in enrichment analysis we found several connections between miRNAs and pathways that may play a role in physiological regeneration or differentiation in endurance sport including BDNF and insulin signaling pathway (Walsh et al., 2020). BDNF is a member of the neurotrophin family, which is a well-known mediator in the development of the nervous system while supporting the survival of neurons and induces neurogenesis (Eyileten et al., 2017, 2020; Gasecka et al., 2020). BDNF acts through several different pathways including the MAPK pathway and PI3K-Akt cascade, which stimulates cell survival (Eyileten et al., 2020). Apart from the nervous system, it is well-known that BDNF can play an important role in glucose/energy homeostasis. Interestingly, many studies provided viable evidence of miRNAs-mediated post-transcriptional regulation of BDNF (Eyileten et al., 2017, 2020). Several human studies and meta-analyses showed that physical exercise leads to elevation of blood BDNF levels (Mrówczyński, 2019; Ruiz-González et al., 2021). Physical exercise induces BDNF secretion which leads to an increase of lactate levels in the blood, and elevation of CV response (Schiffer et al., 2011). If the physical activity is excessive training, increased sympathetic activity can cause platelet activation which contributes to the BDNF release to the blood circulation, as platelets contain the major source of BDNF (Fujimura et al., 2002; Eyileten et al., 2016, 2019; Walsh et al., 2017). In our bioinformatic analysis, among other important pathways also magnesium and guanosine triphosphate were found to be regulated by analyzed miRNAs. Of note, animal studies reported that magnesium can enhance exercise performance *via* ameliorate glucose availability in the brain, muscle and blood circulation by reducing/delaying lactate accumulation in the muscle, and thus reducing fatigue (Zhang et al., 2017). Also free extracellular guanosine 5′-triphosphate (GTP) has been demonstrated to be an improver of myogenic cell differentiation in both mouse and human cell line, which may suggest that guanosine triphosphate may serve as a miRNA-myogenic regulatory factor modulation and thus influence adaptation to physical activity (Pietrangelo et al., 2018).

Interestingly, we found that miR-1-3a was negatively correlated with hs-CRP after completion of the run. More importantly, we found higher miR-1-3p expressions in runners, who finished the race in under 10 h compared to runners who finished over 10 h. MiR-1 is a member of the subgroup of striated muscle-specific or muscle-enriched miRNAs called myomiRs. These molecules are involved in the regulation of muscle development, homeostasis and regeneration, as well as hypoxia/reoxygenation-induced cardiomyocytes apoptosis (Zilahi et al., 2019). It has been suggested that myomiRs show a dose-response correlation at different levels of exercise intensity and duration, and miR-1 expression pattern is dose-dependent with exercise intensity, and other miRNAs such as miR-133a or miR-222 depend on duration of exercise (Ramos et al., 2018). Previously, miR-1 has been reported to respond particularly to aerobic exercise as its concentration increases post-marathon in runners and in young males after high-intensity interval training (HIIT) or vigorous distance-matched exercise (Clauss et al., 2016; Denham and Prestes, 2016). However, specific circulating miR-1 was significantly up-regulated 3 h post exercise but not immediately or shortly after (within <1 h), which concords with our findings as blood samples for analysis were obtained within 30 min after finishing the race (Nielsen et al., 2014; Denham et al., 2018). We previously described that several studies found elevated miR-1-3p in response to endurance training, however, only one study correlated this finding with myoglobin at 24 h after the run (Soplinska et al., 2020; Yin et al., 2020). Moreover, in the current study we found a negative association between miR-1-3p and CRP levels after the 100 km race. There is a temporary rise in blood CRP levels after and during endurance exercise, caused by exercise-induced acute phase response regulated by cytokines, especially IL-6 (Kasapis and Thompson, 2005). Both short-term and long-term endurance sport can induce both anti- and pro-inflammatory responses (Barros et al., 2017). It was also previously reported that CRP level may continuously increase throughout ultra-endurance runs and strongly correlates with distance covered (Kłapcińska et al., 2013). Thus, miR-1-3p may be considered as an indicator of the reparative processes elicited in response to stressful external stimuli. Moreover, it was found that its expression is decreased in myocardial damage induced by epirubicin and that miR-1 negatively modulates the expression of phosphoinositide 3-kinases catalytic subunit alpha (PIK3CA). As a result, downregulation of PIK3CA yields a significant decrease in phosphorylation of protein kinase B (Akt) and mammalian target of rifampicin kinase (mTOR), a key downstream target gene of the PI3K/Akt pathway that can inhibit apoptosis and increase autophagy ameliorating cardiac injury (Wu et al., 2018).

MiR-125a plays an angiogenic role in hypoxia and inflammation in both endothelium and cardiac muscle (Wade et al., 2019). It was shown that the expression of miR-125a-5p was downregulated in ischemic myocardium shortly after myocardial infarction, whereas in another study reported increased circulating expressions of miR-125a-5p among heart failure patients, especially those with reduced ejection fraction (Boštjančič et al., 2012). Further studies reported increased expressions of miR-125a-5p as a response to paroxysmal atrial fibrillation (da Silva et al., 2017). Additionally, other reports indicate a dynamic character in their circulatory presence after myocardial injury (Niculescu et al., 2015; Yuan et al., 2019). As a summary, damaged myocardium may release miR-125a into the circulation. Moreover, *in vitro* studies have shown that endothelial secretion of miR-125a-5p is induced by shear rates which can be also observed during intensive training (Schmitz et al., 2019). This type of training was found to influence miRNAs expression changes as miR-125a-5p was significantly elevated in response to HIIT and thus may be affected by the intermittent nature of HIIT (Schmitz et al., 2019). Indeed, the potential existence of an intensity-dependent threshold for exercise-induced miRNA release from CV cells was reported for other miRNA species in studies comparing moderate intensity continuous (MOD) exercise to HIIT exercise (Sapp et al., 2020). In the current study we found a higher expression of miR-125a-5p in individuals who participated in the 100 km run. The HUNT study revealed that increased levels of miR-125a were associated with low VO_2_ max in male participants, which is an indicator of cardiopulmonary fitness (Bye et al., 2013). In our previous study we also found that the ultramarathon runners with the highest quartile of VO_2_ max had a lower expression of miR-125a-5p (Eyileten et al., 2021). In the current study we observed a negative correlation between miR-125a-5p and maximum lactate concentration. Ultramarathon runners predominantly rely on energy generated in aerobic pathways. Correspondingly, such athletes possess more active oxidative enzymes (Costill and Fox, 1969; Costill et al., 1987). It was previously reported that mean lactate accumulation increases with the running distance covered and, further, that mean running speed is related to lactate concentration (Costill and Fox, 1969; Pollock, 1977; Sjödin and Jacobs, 1981; Rhodes and McKenzie, 1984; Tanaka and Matsuura, 1984; Saltin et al., 1995). Drawn from these findings was the conclusion that marathon runners adjust their running speed to achieve a level of oxygen uptake that minimizes the increase in lactate concentration in the blood, preventing an exponential lactate rise. Tendency of miR-125a-5p to impede aerobic glycolysis and lactate production has been reported in the context of several types of cancer (Huang et al., 2018). However, data on the active and healthy population is very scarce. Cancer cell’s metabolism is unique, in that the majority of glucose is converted to lactate irrespective of the O_2_ availability. This is achieved through the overexpression of several glucose metabolism-related proteins, such as: (a) glucose transporter (GLUT) 1, (b) hexokinase (HK2), and (c) monocarboxylate transporters. They act in canalizing glucose metabolites from catabolic to anabolic processes, resulting in accelerated cell proliferation, migration, and invasion. Notably, other studies pointed to the negative role of miR−125a in the regulation of HK2, a rate-limiting enzyme for glycolysis (Jin et al., 2017; Luo et al., 2020). While the clinical applicability of these findings is yet to be determined, one study suggested miR-125a as a promising molecular target for laryngeal squamous cell carcinoma (LSCC) on the basis that miR-125a suppresses LSCC progression through targeting of HK2 both *in vitro* and *in vivo* analysis (Sun et al., 2017). Additionally, miR-125a-regulated mitochondrial fission is postulated to be a component of cellular energy disorders. Indeed, upregulated mitochondrial fission is believed to impair mitochondrial adenosine triphosphate (ATP) production by reducing electron transport chain activity (Perdiz et al., 2017), resulting in a decrease in glucose consumption and lactate production. These findings reflect those of the present study, advocating for miR-125a being the key regulator of cell energy metabolism. Increase in miR-125a expression reduced autophagy and cell proliferation, while enhancing the apoptotic rate and pro-inflammatory cascade–TNFα, IL-1β, IL-6, and IL-18 were all elevated—through the downregulation of the PI3K/Akt/mTOR signaling pathway. This suggests that PI3K inhibition intensified the ability of miR-125a to harness the inflammatory response *in vitro* through the regulation of the PI3K/Akt/mTOR signaling pathway (Chen et al., 2019). The PI3K/Akt signaling pathway lies at the nexus of numerous biological processes, which include the cell cycle, apoptosis, angiogenesis, and glucose metabolism. PI3K/Akt can thereby regulate the reduction of glycogen synthesis and enhance glycolysis (Xie et al., 2019). Thus, it may be hypothesized that miR-125a-5p inhibits lactate production in high intensity and long-lasting physical activity *via* control of pyruvate synthesis and, with that, control of acetyl-coenzyme A which feeds into the tricarboxylic acid cycle for ATP production. However, additional studies are needed to better explain the role of miR-125a-5p in energy metabolism in endurance sportsmen. Moreover, it can be also hypothesized that the analyzed miRNAs play an adaptive role in ultra-marathon runners as was described for miR-1, miR-125a-5p, and miR-126, also alleviating endothelial cell damage through restoration of autophagic flux by PI3K/Akt/mTOR signaling inhibition (Tang and Yang, 2018; Wu et al., 2018). It is important to note that our previous study evaluated several typical CV-related biomarkers alterations during 100 km-race in elite athletes. We showed that participation in a 100 km ultra-marathon leads to a modest, but significant hs-TnT increase in the majority of runners. Additionally, mean lactate concentration during the race and change in hs-CRP correlated with troponin change (Małek et al., 2020).

Another miRNA- miR-15a may play an important role in glucose metabolism during physical activity. Several *in vivo* and *in vitro* analysis showed that miR-15a regulates the expression of transcription factors which leads to insulin resistance development (Chakraborty et al., 2014). In limited studies miR-15a was found downregulated in patients with type 2 diabetes mellitus (T2DM) compared to non-diabetic subjects (Zampetaki et al., 2010; Jiménez-Lucena et al., 2018). Importantly, our previous bioinformatic analysis pointed out that miR-15a may have an influence on blood coagulation, platelet activation, insulin signaling and glucose metabolism processes (Pordzik et al., 2019). In the current study, we found a negative correlation between miR-15a expression and glucose levels after the race, which is in line with the previous findings. Interestingly, there was no difference in miR-15a expression before and after the run. It can be hypothesized that complex insulin action on peripheral tissues may be associated with miR-15a expression, which may influence other regulatory mechanisms that prevent high glucose level, however, further studies are essential to explain this issue.

Endurance training leads to various changes and processes. It can cause damage of skeletal muscles (Armstrong, 1986) which is, besides i.e. oxidative stress (Duca et al., 2006), one of the factors enhancing training-related inflammation (Kaufmann et al., 2021). Furthermore, high intensity endurance training can result in transient acute volume overload of the atria and right ventricle (Patil et al., 2012). We hypothesize that these phenomena may be related to elevated levels of miR-223 in ultramarathon runners post-race. MiR-223 synthesis is thought to be stimulated by damaged myofibrils and muscle ischaemia. Additionally, the upregulation of miR-223 is correlated with the presence of infiltrating inflammatory cells (Greco et al., 2009; Rangrez et al., 2012; Taïbi et al., 2014), probably due to its essential role in macrophage differentiation and function. MiR-223 can target PBX/knotted 1 homeobox 1 causing macrophage polarization into anti-inflammatory type (Taïbi et al., 2014; Zhou et al., 2015). MiR-223 may also regulate inflammation by inhibiting synthesis and preventing the excess accumulation of NLRP3, a part of inflammasome acting in response to the cellular damage (Taïbi et al., 2014). All of the aforementioned regulatory functions that maintain the balance between inflammatory and anti-inflammatory factors may be beneficial for skeletal muscle regeneration (Cheng et al., 2020). Another suspected function of miR-223, potentially of value for endurance athletes, is its ability to influence metabolic signaling. Indeed, miR-223 may influence the insulin sensitivity of adipose tissue and stimulate GLUT4 expression in cardiomyocytes, which enables increased glucose uptake by those cells (Taïbi et al., 2014). Paralleling this phenomenon is the finding that ablation of miR-223 results in enhanced IFNγ/LPS-induced nitric oxide synthase 2 (NOS2) expression, an enzyme which lies at the nexus of insulin resistance as a potent source of oxidative stress (Deiuliis et al., 2016). Collating these results, it can be suggested that the overexpression of miR-223 in ultramarathon runners is related to metabolic adaptation, skeletal muscle damage, and inflammation.

## Study Limitations

One of the major limitations of our study is that miRNAs expression was assessed in a relatively small group of ultra-marathon runners and with a lack of positive and negative control group comparison. Nonetheless, our cohort of athletes remains one of the largest for which circulating miRNAs have been evaluated, to date. Other limitations are related to the demographic characteristics of our subjects including gender, as the majority of participants were male (87%). As cardiac function and structure measurement after the race and long-term follow-up was unavailable in this study, we are not equipped to draw firm conclusions on long-term effects and we cannot comment whether the changes observed were harmful. Additionally, the present study did not obtain amplification of the hsa-miR-106-5p. This could be explained by low yield of target sequence and influence of additives present in plasma separation tubes which may have affected miRNA quantitation. Indeed, chelator K2EDTA has the ability to interfere with PCR reaction by binding with magnesium chloride, an important source of magnesium for PCR, thereby affecting primer annealing temperature, specificity, and amount of qPCR product (Beekman et al., 2009). The RNA isolation procedure usually removes EDTA contamination, but traces of K2 can persist and affect the amplification process if the amount of miRNAs targets is very low or if primer design is not optimal. This can be improved by choosing NaF/KOx treated tubes, different primers of method of expression analysis (Cacheux et al., 2019). Moreover, we did not have the possibility to assess the miRNAs alterations after a resting period at the recovery phase. Additionally, a 30 min blood collection time frame post the termination of the trial may slightly affect the credibility of our findings. A more comprehensive genome-wide analysis of miRNAs regulated by acute and long-term exercise training is also warranted. Studies acknowledged that differences in health habits and status—diet (Kura et al., 2019), regular physical activity (Barber et al., 2019), and, more broadly, medical history—could produce differences in baseline miRNA expressions between subjects such that results cannot be conclusively attributed to participation in the ultramarathon (Kura et al., 2019). In our analysis we have aimed to perform bioinformatic analysis to determine the miRNAs related to angiogenesis, cardiac muscle function, muscle hypertrophy, coagulation, inflammation, and fibrosis processes. However, extreme physical activity, such as ultramarathon, could affect many organs’ and tissues’ function, not only heart function. Therefore, a future study recruiting more subjects and allowing for propensity score matching between exposure and control groups could clarify the role of intense exercise bouts such as ultramarathon races on miRNA profiles and miRNAs involved in signaling pathways.

## Conclusion

Changes in circulating miRNAs’ expression in response to endurance exercise point to their key role in evaluating specific exercise-induced adaptation. Similarly, this may in turn exert an impact on adaptation to training. The negative correlations between miR-125a-5p and lactate concentrations, and miR-1-3p and CRP further support this hypothesis. However, their protective effects should be further evaluated in this setting. Future studies will be essential in elucidating the impact of extensive and regular physical exercise on selected miRNAs. Overall, our results stand in a context of the need for novel biomarkers to facilitate the diagnostic differentiation of physiological and pathological ends of the cardiac remodeling spectrum in athletes, and the broader promise to catalog the miRNAome to prognostic ends.

## Data Availability Statement

The original contributions presented in the study are included in the article/Supplementary Material, further inquiries can be directed to the corresponding author.

## Ethics Statement

The studies involving human participants were reviewed and approved by the Ethics Committee of the Regional Medical Chamber in Warsaw (no. 52/17). The patients/participants provided their written informed consent to participate in this study.

## Author Contributions

CE, AF, MP, ZW, MM, JS, and SW: writing—original draft preparation. ZW: bioinformatic analysis. CE, MP, LM, SD, SW, and JP: writing—review and editing. CE and MP: visualization. CE, LM, MP, SD, and JP: supervision. CE and ZW: graphical design. CE, MM, AF, and JS: laboratory analysis. All authors contributed to the article and approved the submitted version.

## Conflict of Interest

The authors declare that the research was conducted in the absence of any commercial or financial relationships that could be construed as a potential conflict of interest.

## Publisher’s Note

All claims expressed in this article are solely those of the authors and do not necessarily represent those of their affiliated organizations, or those of the publisher, the editors and the reviewers. Any product that may be evaluated in this article, or claim that may be made by its manufacturer, is not guaranteed or endorsed by the publisher.

## Footnotes

1
https://github.com/wizbionet/wizbionet/blob/master/doc/vignette_wizbionet.md

2
https://www.maratonypolskie.pl/mp_index.php?dzial=3&action=5&code=48684

## Supplementary Material

Supplementary Figure 1 Enrichment analysis results of the selected miRNAs based on bioinformatic analysis.
