# Supplementary figures and images for: Altered Circulating MicroRNA Profiles After Endurance Training: A Cohort Study of Ultramarathon Runners
Source: Front Physiol. 2022 Jan 25;12:792931. doi: 10.3389/fphys.2021.792931 (PMC8824535; doi:10.3389/fphys.2021.792931)

hsa-miR-1

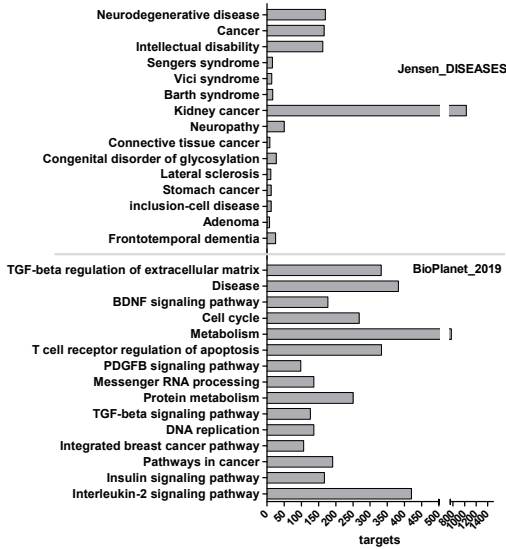

hsa-miR-126

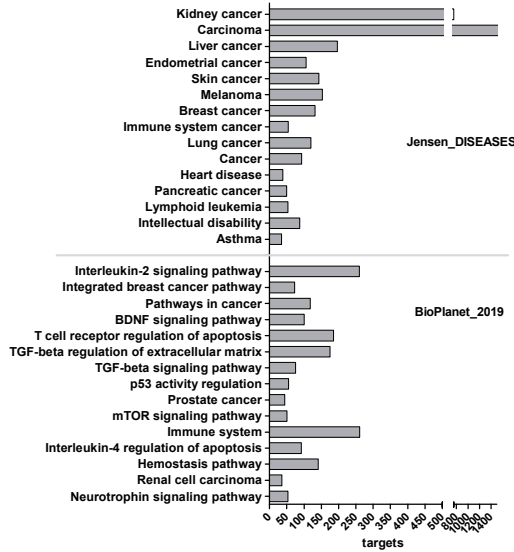

hsa-miR-223

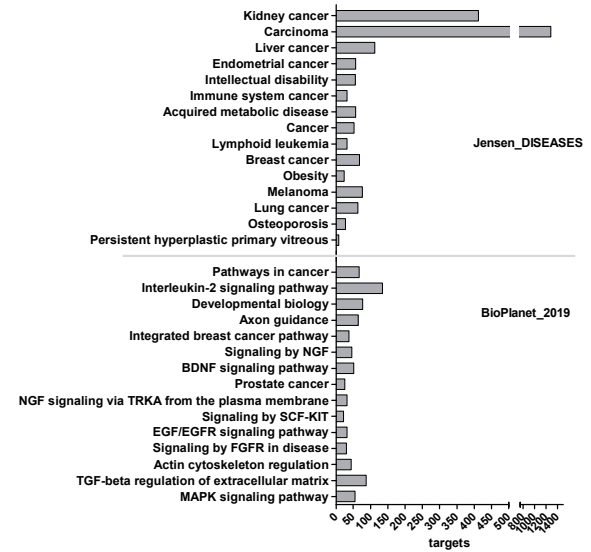

hsa-miR-125a

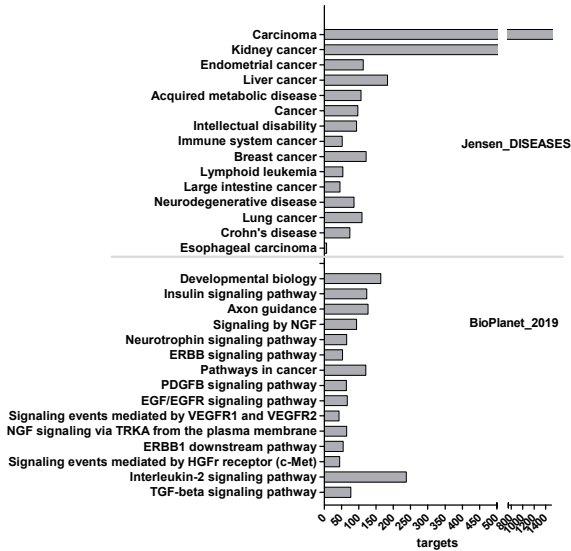

hsa-miR-106a

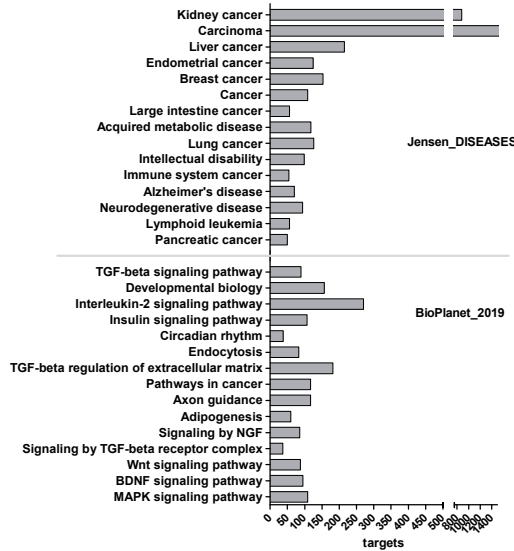

hsa-miR-15b

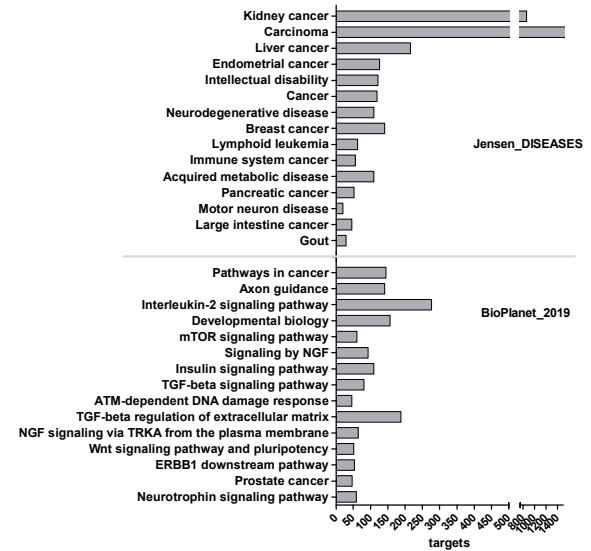

Supplement: Supplementary Figure 1 — Enrichment analysis results of the selected miRNAs based on bioinformatic analysis. [file Data_Sheet_1.PDF]
